# Post-traumatic proximal radioulnar synostosis treated with (modified) reverse Sauvé–Kapandji procedure: a case report

**DOI:** 10.1016/j.xrrt.2025.04.007

**Published:** 2025-05-19

**Authors:** Minke Boeijenga, Leo Geeraedts, Charlotte M. Lameijer

**Affiliations:** Department of Trauma Surgery, Amsterdam UMC, Location AMC, Amsterdam, The Netherlands

**Keywords:** Case report, Proximal radioulnar synostosis, Heterotopic ossification, Monteggia fracture-dislocation, Synostosis excision, Reverse Sauvé-Kapandji

Post-traumatic synostosis of the proximal radioulnar joint (PRUJ) is a rare complication that can occur after fractures of the elbow and forearm.^4^^,^^16^ Due to ossification of the radius and ulna, patients may experience difficulty or complete loss of forearm rotation. This can have major functional impact on daily activities and, consequently, on the quality of life. Management of this PRUJ synostosis remains controversial, as there is no therapeutic consensus yet.^6^ In vital and active patients, surgical excision of the synostosis is the prevalent choice of treatment.^4^ Numerous case series and case reports about surgical treatment options and outcomes have been published; however, studies with highly detailed procedures regarding proximal radioulnar synostosis are lacking. In this case report, we present a case of post-traumatic proximal radioulnar synostosis following an initial open Monteggia fracture–dislocation, treated with the reverse Sauvé–Kapandji procedure by leaving the synostosis partially intact along with a 3-cm resection of the radial neck to regain pronation and supination.

## Case presentation

**Figure 1:**
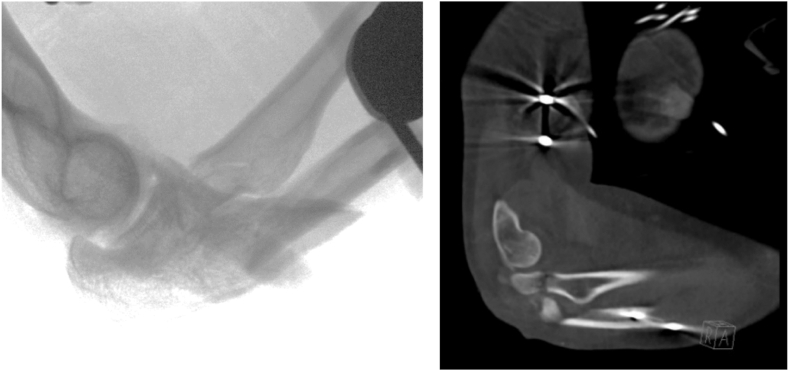
Imaging of the right elbow showing extensive comminuted fracture of the radial neck with displacement, comminuted fracture of the proximal ulna, and fracture of the coronoid process. Artefacts of the external fixator in the humerus are visible. Preoperative X-ray imaging not available due to the extent of other injuries.

**Figure 2:**
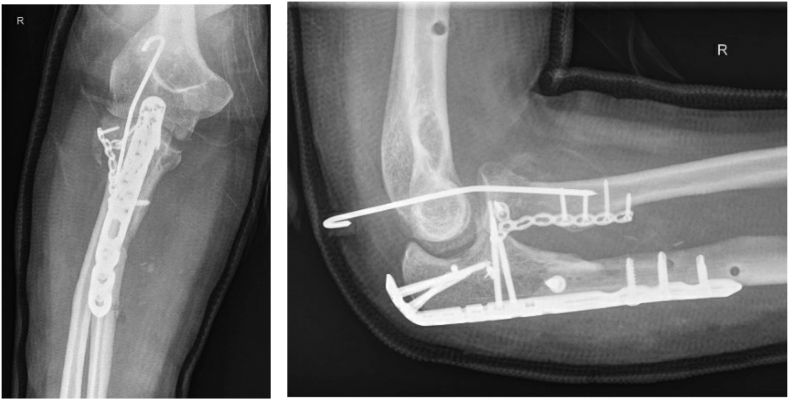
Plate osteosynthesis of the olecranon, plate osteosyntheses, and K-wire fixation of the proximal radius (K-wire was removed 4 weeks postoperatively).

**Figure 3:**
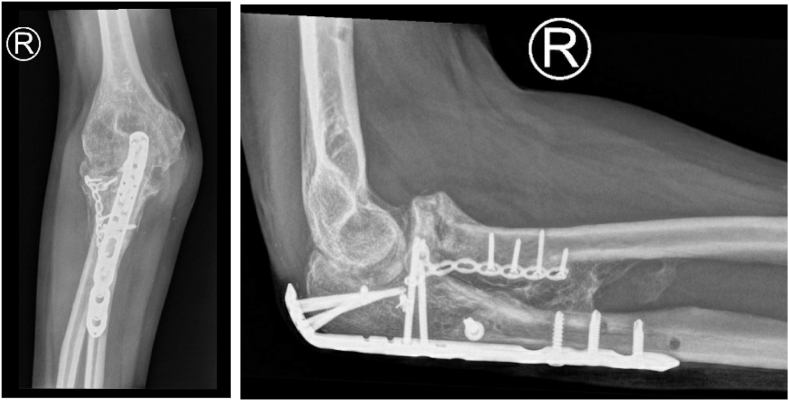
Extensive post-traumatic proximal radioulnar synostosis.

A 43-year-old female presented to the emergency department after a fall from height (18 m) with polytrauma, including a pelvic fracture, subdural hematoma, frontal head laceration, calcaneal fracture of the right foot, olecranon fracture of the left elbow, and a fracture–dislocation of the right elbow. In addition, a Monteggia fracture–dislocation (Bado type IV) of the right elbow was diagnosed with diminished distal radial pulses and a cold right hand (Fig. 1). Hence, the fracture and wound were débrided in the operating room, neurovascular functions were secured, and the fracture was stabilized with an external fixator. After multiple surgeries, including fixation of the pelvic fracture, indomethacin was administered for 2 weeks in attempt to prevent infection and heterotopic ossification (HO). Within these 2 weeks, the right elbow was surgically treated with open reduction and internal fixation (Fig. 2) at which a temporary transfixation of the radial head was achieved with a K-wire, due to persistent instability. An upper arm cast was applied to immobilize the joint for 3 weeks, followed by a hinged brace for 3 weeks (after removal of the K-wire). The patient was discharged from the hospital 2.5 months after the initial trauma and she was regularly seen at the outpatient clinic. In the following months, range of motion (ROM) of the right elbow deteriorated in pronation/supination from 10°/0°/10° to 0°/0°/0°, but maintained a relatively consistent flexion/extension arc of 140°/40°/0° (Table I). Radiographs and computed tomography images showed radioulnar synostoses between the proximal radius and ulnar shaft and between the radial head and coronoid process (Fig. 3), corresponding to a type 3 synostosis according to the Vince and Miller classification.^19^ Fractures from the initial trauma had united and the osteosynthesis material was still correctly in place. Due to the invalidating symptoms of the radioulnar synostosis, the patient consented to a third surgery.

**Table I:** Functional outcomes during follow-up.

PROM				Active ROM			
PROMIS UE v2.0 SF-7a	DASH	Q-DASH	MEPS	Flexion (°)	Extension (°)	Pronation (°)	Supination (°)
6 mo after trauma							
N/A	N/A	N/A	N/A	140	40	10	10
Before surgery							
23.9	N/A	N/A	N/A	140	40	0	0
Perioperatively							
N/A	N/A	N/A	N/A	N/A	N/A	70	70
4 mo after surgery							
30.5	N/A	N/A	N/A	130	20	80	40
14 mo after surgery							
40.8	6.67	6.82	95	140	30	85	45

*PROM*, patient-reported outcome measure; *ROM*, range of motion; *PROMIS UE v2.0 SF-7A*, Patient-Reported Outcomes Measurement Information System Upper Extremity version 2.0 Short Form 7A; *(Q-) DASH*, (Quick) Disability of the Arm, Shoulder, and Hand questionnaire; *MEPS*, Mayo Elbow Performance Score.

### Operative technique

**Figure 4:**
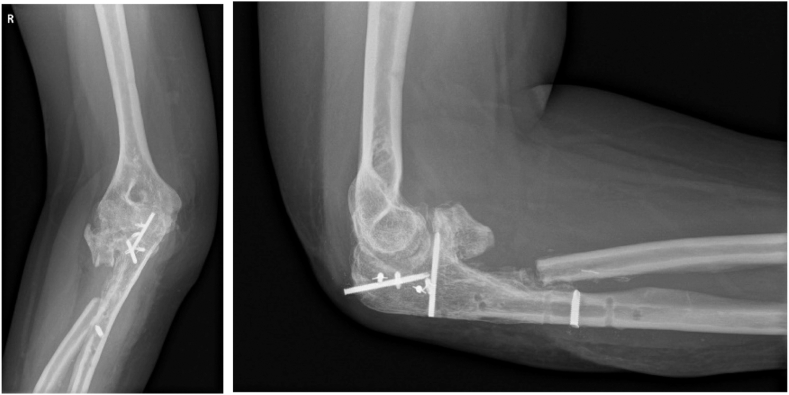
Postoperative imaging after the reverse Sauvé–Kapandji procedure.

A reverse Sauvé–Kapandji procedure of the right elbow was performed. The patient was put under general anesthesia with a tourniquet on the upper arm in prone position. The previous scar was incised, and the Boyd approach was used to dissect to the ulnar shaft. After removing the osteosynthesis material from both the proximal ulna and radius, dissection continued over the ulnar shaft proximally to the synostosis. As it is mandatory to get to the proximal radius in the Boyd approach, the insertion of the lateral collateral ligament (LCL) was released. Caution to protect the posterior interosseous nerve was taken into account while removing the synostosis between the radius and ulna. In anticipation of full PRUJ and radiocapitellar instability, the most proximal synostosis between the radial head and coronoid process was left intact, maintaining radiocapitellar stability. A 3-cm section of the radial neck was resected with an oscillating saw and thereby the reverse Sauvé–Kapandji was executed (Fig. 4). The LCL was reinserted to the ulna with the aid of 2 bone anchors (Mitek GII QA+ with orthocord). Perioperative testing showed excellent ROM of the elbow, with flexion/extension of 140°/40°/0° and pronation/supination of 70°/0°/70°. Again, indomethacin was administered for 10 days postoperatively to diminish the risk of HO. A compression bandage was applied for 48 hours after surgery. Elbow rehabilitation with physical therapy was started as soon as possible to maintain the ROM achieved during surgery. However, varus stress was limited during 6 weeks due to the LCL reinsertion.

### Functional outcome

The patient received physical therapy at home twice a week and she was followed up regularly at the outpatient clinic, including radiographic imaging. No complications were reported after surgery. After 4 months of follow-up, the patient had slightly improved pronation of the right elbow and she regained the ability to perform daily activities. Nevertheless, according to the patient, supination had deteriorated over time due to inactivity and lack of exercise. After more than one year of follow-up, the patient reported acceptable ROM and a greatly improved Patient-Reported Outcomes Measurement Information System Upper Extremity v2.0 score of 40.8, which is well above the reported minimal important change threshold of this questionnaire.^5^^,^^11^ The patient was pleased with the overall functioning of the right upper extremity in daily life (Table I).

## Discussion

Complex elbow injuries are characterized by dislocation of the elbow with concomitant fracture of one or more of the osseous stabilizers of the joint. Management of these complex injuries is challenging and is often accompanied with poor functional outcomes, including persistent instability, stiffness, and post-traumatic arthrosis.^20^ A less frequent but troublesome complication of these injuries and/or their subsequent surgical procedures is HO, which is the sequela of ectopic bone remodeling in or around the joint.^13^^,^^14^ Due to HO, a cross-union between the proximal ulna and radius may develop, also known as post-traumatic proximal radioulnar synostosis (PPRUS).^1^^,^^6^^,^^14^ The occurrence of HO formation has been reported in 6.6% of proximal radius and ulna fractures,^3^ which puts patients with Monteggia fracture–dislocations at higher risk of developing PPRUS. The abnormal ossification between the radius and ulna may result in limitation of axial forearm rotation, eventually resulting in a total loss of active pronation and supination, equivalent to our case.^17^

**Figure 5:**
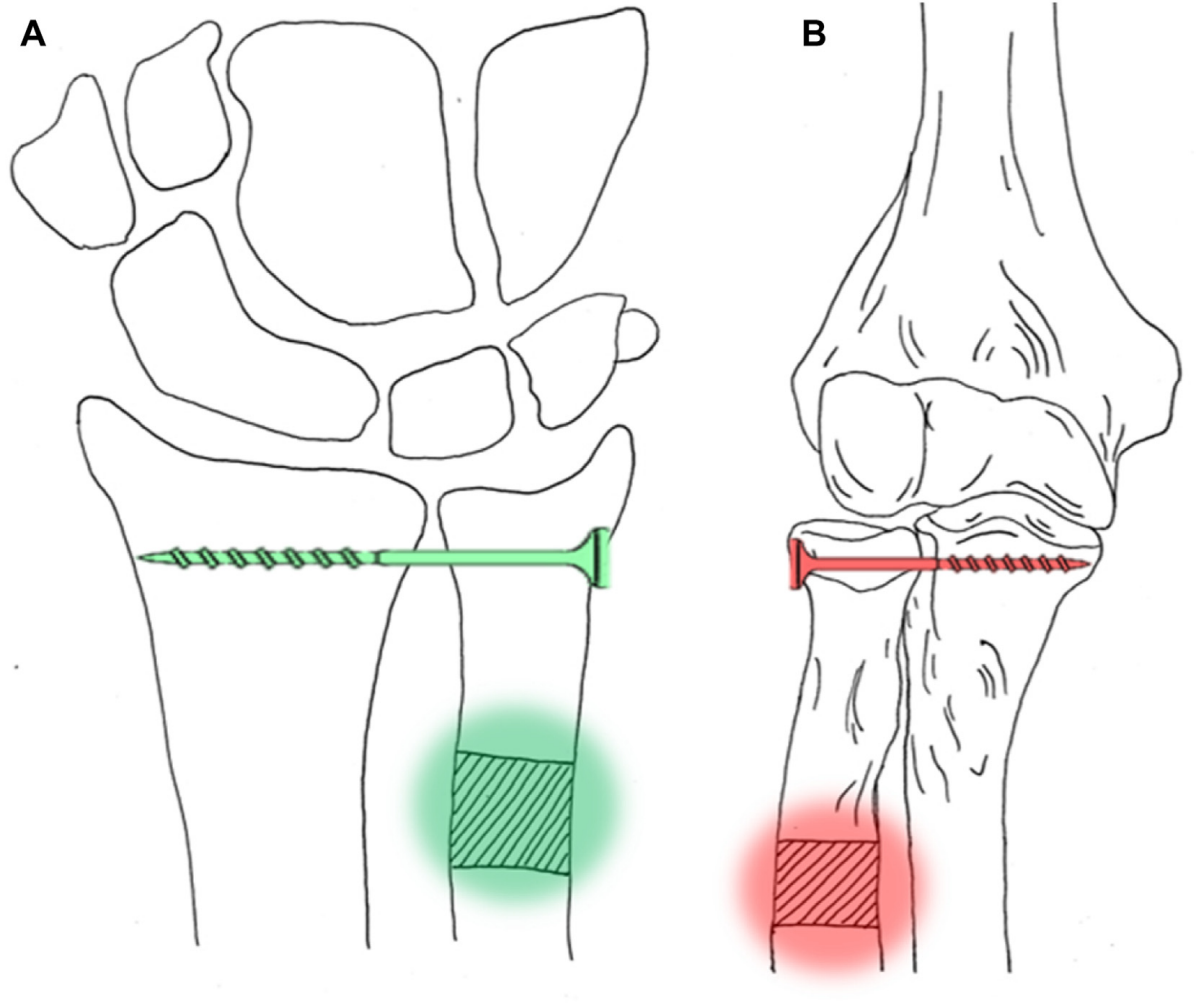
Overview of the principles of the Sauvé–Kapandji and reverse Sauvé–Kapandji procedures [own material], (**A**) original Sauvé–Kapandji procedure with arthrodesis of the distal radial ulnar joint and partial osteotomy of the distal ulna and (**B**) reverse Sauvé–Kapandji procedure with arthrodesis of the PRUJ and partial osteotomy of the proximal radial shaft. *PRUJ*, proximal radioulnar joint.

Besides the fact that PPRUS is rare, there is no clear consensus on optimal surgical treatment. Numerous surgical treatment options have been described in literature with varying functional outcomes (Table II).^7^, ^8^, ^9^, ^10^^,^^12^ These surgical treatment options include complete synostosis excision and partial radial shaft resection, also known as the reverse Sauvé–Kapandji procedure. In 1936, Gonzague Sauvé and Mehmed Kapandji first reported a surgical technique in which arthrodesis of the distal radioulnar joint in combination with a partial ulnar osteotomy proximal to the arthrodesis was performed to create a pseudoarthrosis of the distal ulna to maintain or improve forearm rotation, currently known as the Sauvé–Kapandji procedure (Fig. 5).^2^^,^^15^^,^^18^ The studies reported by Jimenez, et al, Kamenini, et al and Kamrani, et al show promising results for a surgical procedure involving the partial resection of the radial shaft distal to the synostosis and leaving the synostosis in place, which can also be described as the proximal or reverse Sauvé–Kapandji procedure.^8^, ^9^, ^10^

**Table II:** Literature overview on post-traumatic proximal radioulnar synostosis.

Authors	Initial injury	Surgical technique	Postoperative functional outcomes
Giannicola, et al 2020^7^	1. Elbow fracture-dislocation	Synostosis excision and proximal radial resection with anconeus interposition	Mean functional outcomes: 20° extension, 136° flexion, 63° pronation, 60° supination
	2. Monteggia-like lesion	Synostosis excision, radial head arthroplasty, and LCL reinsertion	
	3. Monteggia-like lesion	Synostosis excision, radial head resection, and anconeus interposition	
	4. Complex articular fracture of distal humerus	Synostosis excision and hardware removal	
	5. Terrible triad	Joint débridement with synostosis excision, radial head arthroplasty, and LCL reinsertion	
	6. Terrible triad	Joint débridement with synostosis and HO excision	
	7. Elbow dislocation with coronoid and proximal radius fractures	Proximal radial diaphyseal resection with anconeus interposition and bone wax	
	8. Monteggia-like lesion	Synostosis excision and hardware removal	
	9. Radial head fracture with dislocation	Joint débridement with synostosis and ulnohumeral HO excision	
	10. Monteggia-like lesion	Synostosis excision	
	11. Monteggia-like lesion	Débridement with synostosis and HO excision, radial head arthroplasty, LCL reinsertion, and hardware removal	
	12. Radial head fracture with dislocation	Synostosis excision, radial head arthroplasty removal, anconeus interposition, and LCL retensioning	
Jimenez, et al 2018^8^	Case 1: open elbow fracture-dislocation Type II of Gustilo and Anderson	Reverse Sauvé–Kapandji, resecting 1-cm section of the radial shaft. Kocher approach. Synostosis left in place	30° extension, 125° flexion, 75° pronation, 40° supination
	Case 2: non-displaced olecranon and radial head fracture	Reverse Sauvé–Kapandji, resecting 1-cm section of the radial shaft. Synostosis left in place	0° extension, 130° flexion, 35° pronation, 40° supination
Kamineni, et al 2002^9^	1. Fracture-dislocation of elbow, fractures of radial head and coronoid	10 mm of radius resected (distal to tuberosity)	−75° extension, 90° flexion, 90° pronation, 25° supination
	2. Fracture of the proximal ulna	8 mm of radius resected (distal to tuberosity), synostosis takedown, capsulotomy, excision of olecranon tip	−30° extension, 135° flexion, 90° pronation, −30° supination
	3. Fracture-dislocation of elbow	7 mm of radius resected (distal to tuberosity), anterior capsulectomy, removal of metalwork	−30° extension, 130° flexion, 70° pronation, 45° supination
	4. Fracture of radial head and neck	10 mm of radius resected (distal to tuberosity), anterior capsulectomy	−45° extension, 135° flexion, 75° pronation, 30° supination
	5. Open comminuted fracture of proximal olecranon, radial head, and coronoid	5 mm of radius resected (distal to tuberosity)	−20° extension, 130° flexion, 90° pronation, 85° supination
	6. Fracture-dislocation of elbow	10 mm of radius resected (between head and tuberosity), ectopic bone excised anteriorly and posteriorly	−45° extension, 135° flexion, 20° pronation, 20° supination
	7. Fracture of elbow	5 mm of radius resected (distal to tuberosity)	0° extension, 100° flexion, 35° pronation, 45° supination
Kamrani, et al 2014^10^	15 patients with posttraumatic radioulnar synostosis	Resection of 1 cm of the radial diaphysis distal to the synostosis	Mean functional outcomes: Flexion/extension arc: 81° ± 33° Pronation/supination arc: 101° ± 45°
Khadka M., et al 2023^12^	Monteggia fracture-dislocation	Excision of excessive fibro-osseous connection in the proximal radius and ulna and removal of the implant	Near full pronation and supination

*LCL*, lateral collateral ligament; *HO*, heterotopic ossification.

In the treatment of our patient, the radioulnar synostosis was partially excised by resecting 3 cm of the radial neck. The radial head, which was fused with the olecranon, was left in place to maintain stability and function as arthrodesis (similar to the fusion of the distal radial ulnar joint in the original Sauvé–Kapandji procedure). In our case, a larger part of the radial shaft was resected than that has been described in previous literature. However, due to the high extent of ossification, this rather large excision fragment had to be removed and was therefore considered to be the best patient-specific treatment. Nevertheless, perioperatively and postoperatively, acceptable ROM was achieved and the patient regained pronation and supination of the forearm.

This case report illustrates the challenging treatment of PPRUS, in which a modified approach of the reverse Sauvé–Kapandji procedure was performed, resulting in improved clinical results. The lack of surgical consensus and variation in PPRUS emphasize the importance of extensive and detailed surgical planning and our solution may be an addition to the armamentarium of a dedicated upper extremity/elbow surgical team.

## Conclusion

Even though post-traumatic proximal radioulnar synostosis is of rare occurrence, physicians should be aware of this PRUJ disorder when patients present with deteriorating forearm rotation after complex elbow trauma or surgery. Treatment requires high level of surgical skills and a thorough understanding of pathoanatomy. This case report demonstrates good functional outcomes following the reverse Sauvé–Kapandji procedure after initially suffering an open Monteggia fracture–dislocation of the elbow and it is therefore considered to be a reliable surgical approach in the treatment of PPRUS.

## Disclaimers

Funding: The authors declare they did not receive any financial support or other funding related to writing this article.

Conflicts of interest: The authors, their immediate families, and any research foundation with which they are affiliated have not received any financial payments or other benefits from any commercial entity related to the subject of this article.

Patient consent: Obtained.
